# Genome damage accumulated in seed ageing leads to plant genome instability and growth inhibition

**DOI:** 10.1042/BCJ20230006

**Published:** 2023-04-04

**Authors:** Wanda M. Waterworth, Christopher E. West

**Affiliations:** Centre for Plant Sciences, University of Leeds, Woodhouse Lane, Leeds LS2 9JT, U.K.

**Keywords:** genome stability, germination, seed

## Abstract

Successful germination and seedling establishment are important determinants of crop yields and plant survival in natural environments. Germination potential is compromised by suboptimal environmental conditions that result in seed ageing and high levels of genome damage. However, the mutagenic and growth inhibitory potential of DNA damage accumulated in seeds on subsequent seedling growth remains largely unknown. Arabidopsis seeds deficient in the chromosomal break repair factors DNA LIGASE 4 and DNA LIGASE 6 exhibited hypersensitivity to the effects of natural ageing, with reduced germination vigour and seedling biomass relative to wild type seed. Here, we identify that aged Arabidopsis seed display elevated levels of programmed cell death (PCD) in the root meristem which persists into seedling establishment, with higher levels of cell death in lines deficient in DNA double strand break repair. Reporter lines determined the effects of seed ageing on mutation levels and intrachromosomal recombination frequencies. Seed deterioration resulted in strikingly elevated levels of frameshift mutations and genome instability in germinated seedlings. Thus, elevated levels genome damage incurred in the seed stage of the plant life cycle potentially impacts significantly on subsequent plant development. Furthermore, the mutagenic effects of seed ageing has potentially long-term implications on the genome stability of plant populations and ecosystem fitness. Collectively, we identify genome damage accumulated in suboptimal quality seed impacts on subsequent plant growth and genome stability, with associated implications for crop yields and plant survival under changing climates.

## Introduction

Seed longevity is an important factor which determines crop yields, and plant survival in natural ecosystems. Desiccation tolerant (orthodox) seed undergoes programmed maturation drying following development and maturation on the mother plant. Low levels of cellular metabolism in the quiescent embryo of the desiccated seed function to prolong seed viability. The transition from quiescent seed to germinated seedling is a critical stage of the plant lifecycle that is particularly vulnerable to environmental perturbation [1]. The low activity of cellular maintenance pathways during extended periods of quiescence is associated with deterioration (ageing) of cellular structures and biological macromolecules, including nucleic acids and proteins [2–5]. This accumulation of cellular damage is exacerbated by suboptimal environmental conditions including drought and high temperatures during seed development and subsequent quiescence, impacting on seed quality [6]. Loss of seed viability is preceded by decreasing germination vigour, characterised by declining rapidity and uniformity of germination and increased susceptibility to biotic and abiotic stresses. Furthermore, the resulting seedlings display increased vulnerability to environmental stresses such as drought, predation, pathogen attack and weed competition [7].

The ability of the seed to withstand or repair cellular damage upon imbibition is crucial to the maintenance of seed germination vigour and viability [8] and the decline in vigour is accompanied by an extended period of genome repair [9]. In Arabidopsis, germination, which is completed upon emergence of the radicle from the seed coat [10], coincides with the initiation of cell cycle activity including DNA replication and cell division [11]. DNA damage accumulated in the quiescent embryo must be repaired prior to cell division in order to minimise mutagenesis and inhibition of seedling growth and development [12]. Cellular DNA damage response pathways and DNA repair activities are initiated early in seed imbibition, before cell cycle activity initiates [3, 9]. DNA double strand breaks (DSBs) represent highly cytotoxic forms of DNA damage, potentially resulting in chromosome fragmentation, loss of genetic information and cell death. Repair of DSBs is important to seed quality illustrated by the markedly reduced vigour and viability in seed germination under stress displayed by Arabidopsis mutants deficient in DSB repair factors DNA LIGASE 4 (LIG4) and DNA LIGASE 6 (LIG6) [3]. LIG4 and LIG6 operate in pathways of non-homologous end joining (NHEJ) that mediate sequence-independent repair of DSBs, with LIG4 catalysing the final step of the canonical NHEJ pathway initiated by the DNA-end binding protein complex of KU70 and KU80 [13]. These mutant lines are viable and display wild type phenotypes under standard growth conditions, but are hypersensitive to treatments that result in chromosomal breaks, including X-irradiation [14].

Rapid fluctuations in environmental conditions during seed maturation and quiescence arising from climate change may significantly impact on seed quality through increasing genome damage at the seed stage of the plant lifecycle. In addition, environmental stresses result in decreased genome stability and elevated recombination frequencies [14, 15]. Particularly high levels of genotoxic stress are associated with the transition from desiccated seed to germinated seedling, relative to other stages of the plant lifecycle [8]. However, the impact of genome damage in seeds on the subsequent growth and genome stability of the germinated seedling is unknown. Furthermore, stresses experienced during vegetative growth can result in mutations that are passed on to progeny, as meristems give raise to reproductive tissues late in plant development [15]. As a result, genome maintenance mechanisms in plant cells are important not only for growth and development, but also in preserving the longer term stability of plant germplasm at the level of populations and species. Previously we demonstrated that experimental application of stresses to seeds, including X-irradiation or accelerated ageing (at 35°C and 83% relative humidity), resulted in PCD in early post-germination [16]. We hypothesised that similar effects occur in natural seed ageing and that cell death persists throughout seedling establishment, indicative of genome instability carried over from DNA damage incurred in the seed. Here, we shown that natural seed ageing over ten years results in prolonged DNA damage responses that persist into the first week of post-germinative seedling growth. Furthermore, we find that the increased levels of genotoxic stress incurred in aged seeds result in increased spontaneous recombination and mutation rates in the genome and thereby negatively influence plant genome stability. The effects of rapidly changing climates on seed quality could therefore significantly impact on the genome stability and mutational frequency of plant populations in the natural environment.

## Results

Our previous work found that seeds were surprisingly resistant to X-ray induced DNA damage [16]. In particular, seeds maintained post-germinative growth after irradiation, with levels of programmed cell death (PCD) that were both delayed and attenuated relative to irradiated seedlings. This led to the hypothesis that delayed responses to DNA damage incurred naturally during seed ageing could have negative effects extending beyond germination, resulting in reduced genome stability, increased cell death and inhibition of early plant growth. These hypotheses were tested through analysis of seedlings germinated from seeds aged naturally or by accelerated ageing.

### Naturally aged Arabidopsis *lig6 lig4* mutant seed display reduced germination vigour

**Figure 1.**
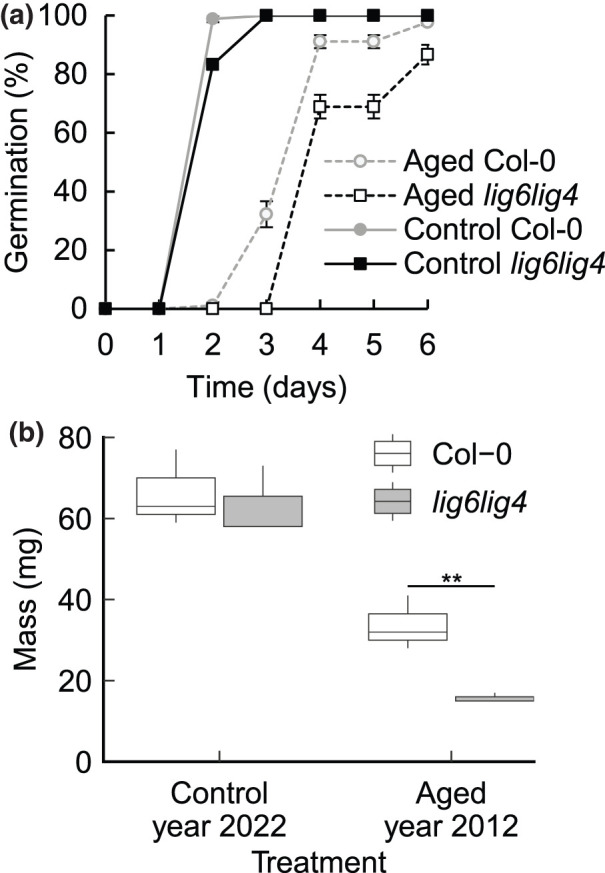
Naturally aged *lig6 lig4* mutant seed display reduced germination and seedling vigour. (**a**) Germination vigour of naturally aged *lig6 lig4* mutant seed is reduced compared with wild type. Analysis of germination vigour of 2021 and 2021 harvests of wild type and *lig6 lig4* mutant seed. Seeds were plated and stratified at 4°C for 48 h before transfer to a growth chamber at 22°C and seeds scored for radical emergence. Standard error of the mean of three replicates of 30 seeds. (**b**) Fresh weight biomass of seedlings germinated on from unaged and aged wild type and *lig6 lig4* mutant 14 days post stratification. Seeds were plated onto MS plates and stratified at 4°C for 48 h before transfer to a growth chamber at 22°C and seedlings harvested at 14 days. Error bars are SEM of three replicates of 10 plants (** *P* < 0.01, *t*-test).

Our previous analysis of DSB-repair deficient Arabidopsis *lig6 lig4* mutant seed established the requirement for chromosomal break repair in seed longevity, revealed through the hypersensitivity of the mutant lines to accelerated ageing [3]. Here we analysed the germination performance of naturally aged wild type and DSB repair deficient seed lots to determine the roles of genome repair in the longevity of seeds after 10 years’ storage under ambient conditions. High quality seeds (2020 harvest) of both wild type Arabidopsis (Col-0) seeds and *lig6 lig4* lines displayed no significant differences in germination (Figure 1a). Naturally aged seed (2012 harvest) of both the mutant and wild type seed lots displayed reduced germination vigour relative to unaged counterparts. However, comparison of the germination of aged wild type and NHEJ mutant lines revealed that the DSB repair deficient lines displayed significantly reduced vigour (mean germination time 3.8d (Col-0) and 4.4d (*lig6 lig4*), *P* < 0.01) and viability (98% Col-0, 87% *lig6 lig4*, *P* < 0.05).

### Deficiency in NHEJ impacts on seedling development

We next evaluated the impact natural seed ageing on subsequent seedling development of lines deficient in chromosomal break repair. The biomass of seedlings grown on half strength Murashige and Skoog media for two weeks post-stratification from unaged Col-0 and *lig6 lig4* mutant seed was similar, with no significant difference between the lines (Figure 1b). However, the biomass of seedlings derived from aged *lig6 lig4* was significantly lower that aged wild types (*P* < 0.01), demonstrating the requirement for effective chromosomal break repair in germination for robust seedling establishment. Furthermore, these results indicate that genome damage sustained in the seed stage of the plant lifecycle can significantly impact on subsequent plant growth and development.

### Arabidopsis *ku80* mutant seed display an elevated incidence of PCD which persists in seedling growth

**Figure 2.**
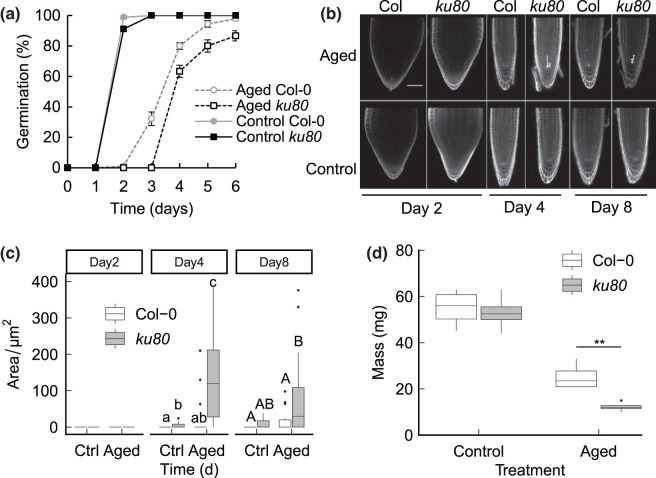
*ku80* mutant seed display elevated incidence of PCD which persists into seedling growth. Germination and ageing-induced cell death in Col-0 and *ku80* mutant lines. Germination of *ku80* mutant seed is hypersensitive to aging. Germination of *ku80* seeds was analysed after accelerated aging at 35°C and 83% RH relative to unaged control seed. Seeds were stratified at 4°C for 2 days before transfer to 23°C 16 h day and scored for radicle emergence each day post-stratification. (**a**) Germination of aged and unaged Col-0 and *ku80* mutant lines. Error bars represent the SEM of the mean of three replicates of 30 seeds. (**b**) PCD in wild-type and DNA repair *ku80* mutant seed was analysed by viability staining. Embryos were isolated from aged seeds for 10 days and control (unaged) seeds and PCD assayed at the time point indicated post-germination. Roots analysed by PI staining and confocal microscopy. Bar is 50 µm. (**c**) PCD was quantified as the area of cell death in aged seeds. Ctrl: control (unaged) seeds. Aged: seeds aged for 10 days. Significance groups are indicated by letters (*P* < 0.05, *n* > 10 per treatment, Kruskal–Wallis with Bonferroni post hoc correction for multiple tests). (**d**) Fresh weight biomass of seedlings germinated on from unaged and aged wild type and *ku80* mutant 14 days post stratification. Seeds were plated onto MS plates and stratified at 4°C for 48 h before transfer to a growth chamber at 22°C and seedlings harvested at 14 days. Error bars are SEM of three replicates of 10 plants (** *P* < 0.01, *t*-test).

Accelerated ageing is a widely used procedure which simulates the natural ageing process by incubating seeds at elevated temperature and relative humidity (RH) (Rajjou 2008). We used accelerated ageing of Arabidopsis lines deficient in the non-homologous end joining (NHEJ) factor KU80, which functions in an early step of DSB repair by binding and stabilsing the DNA end, in addition to recruiting repair factors [13]. As previously reported for KU70, the protein partner of KU80 [16], *ku80* mutants displayed hypersensitivity to accelerated ageing for 10 days at 35°C and 83% relative humidity [16] (Figure 2a). The persistence of ageing-induced genome instability and cell death in subsequent seedling growth was investigated by quantification of dead cells in the root meristem using propidium iodide (PI) staining and confocal microscopy for up to 8 days post-stratification. At two days post-stratification unaged high quality seeds had germinated, while aged seeds exhibited delayed germination (Figure 2a). Cell death was not apparent in either aged or control seeds at this time point (Figure 2b,c). However, at 4 days post-stratification aged seeds germinated and elevated levels of cell death were observed consistently in the *ku80* mutant line and sporadically in wild type seedlings, consistent with induction of PCD by genome damage accumulated in aged seeds. Significantly, we found high levels of ageing-induced cell death at 8 days post-stratification in both wild type and DNA repair mutant seeds (Figure 2b,c). Seedling biomass was reduced in both wild type and *ku80* mutants after ageing, but the mutant lines displayed significant hypersensitivity to the ageing treatment (Figure 2d). These results are consistent with the persistence of DNA damage accumulated in suboptimal seed lots into seedling growth, and supports the hypothesis that ageing induced genome damage negatively affects seedling growth and development.

### Analysis of mutagenesis in seeds

**Figure 3.**
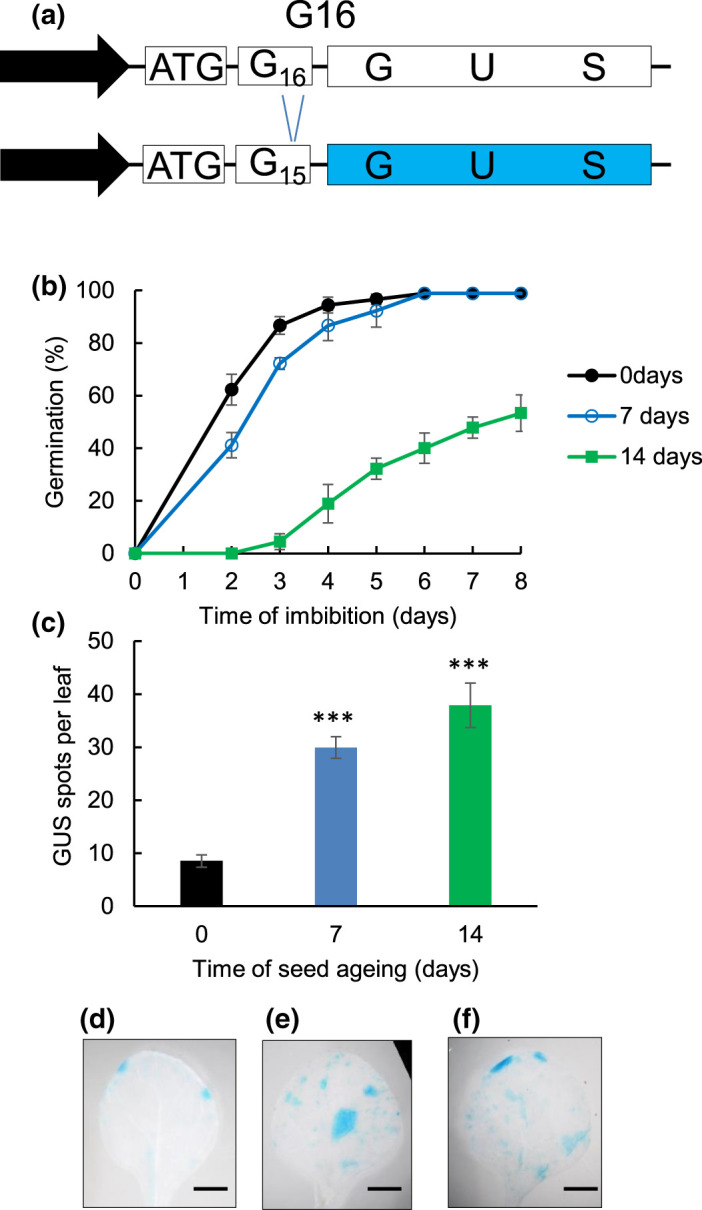
Effects of accelerated ageing on G16 microsatellite frequency. Microsatellite stability was assessed using GUS preceded by 16 guanine residues that disrupt the reading frame of the reporter (**a**) Nucleotide insertion or deletion can lead to restoration of the GUS reading frame and GUS activity. (**b**) Germination performance of G16 microsatellite reporter line after 0 day, 7 days and 14 days accelerated ageing at 35°C and 82%. Seeds were plated and stratified at 4°C for 48 h before transfer to an environmental growth chamber at 22°C and seeds scored for radicle emergence each day post-imbibition. Error bars show the standard error of the mean of three replicates of 50 seeds. (**c**) Frequency of spots on abaxial surface of first true leaves in plants germinated from G16 reporter line after 0 day, 7 days and 14 days accelerated ageing at 35°C and 82%. Seeds were stratified at 4°C for 48 h before transfer to soil. (**d**–**f**) Representative image of mutations frequencies of seedlings germinated from seeds after accelerated ageing for (**d**) 0 days, (**e**) 7 days and (**f**) 14 days.

Increased levels of genome stress can arise from suboptimal environmental conditions during seed development whilst attached to the mother plant or storage and is associated with increased frequencies of chromosomal abnormalities [12]. We therefore evaluated the impact of seed deterioration on the accumulation of mutations in seedlings after accelerated ageing using the G16 reporter line in which frameshift mutations in a run of 16 guanidine nucleotides (a microsatellite) restore translation of a downstream *GUS* reporter gene [17] (Figure 3a). Mutation frequencies were quantified in the first true leaves in seedlings at an equivalent developmental stage germinated from aged seeds and controls. The first two true leaves represent new cell division arising from the embryonic meristem from within the seed. Accelerated ageing for 7 days resulted in reduced seed vigour but little loss of final viability, whereas 14 days ageing reduced viability to ∼50% (Figure 3b). Histochemical staining was used to determine the number of GUS-positive clonal sectors in control and aged seeds (Figure 3c–f). Ageing for 7 days was associated with a 3-fold increase in frameshift mutations in seedlings (Figure 3c), whilst surviving seedlings germinated from seed lots deteriorated for 14 days exhibited a 4-fold increase in blue sectors (Figure 3c–f). This indicates that seed deterioration is associated with progressively increased mutagenesis in the resulting seedling.

### Effects of seed ageing on genome stability

**Figure 4.**
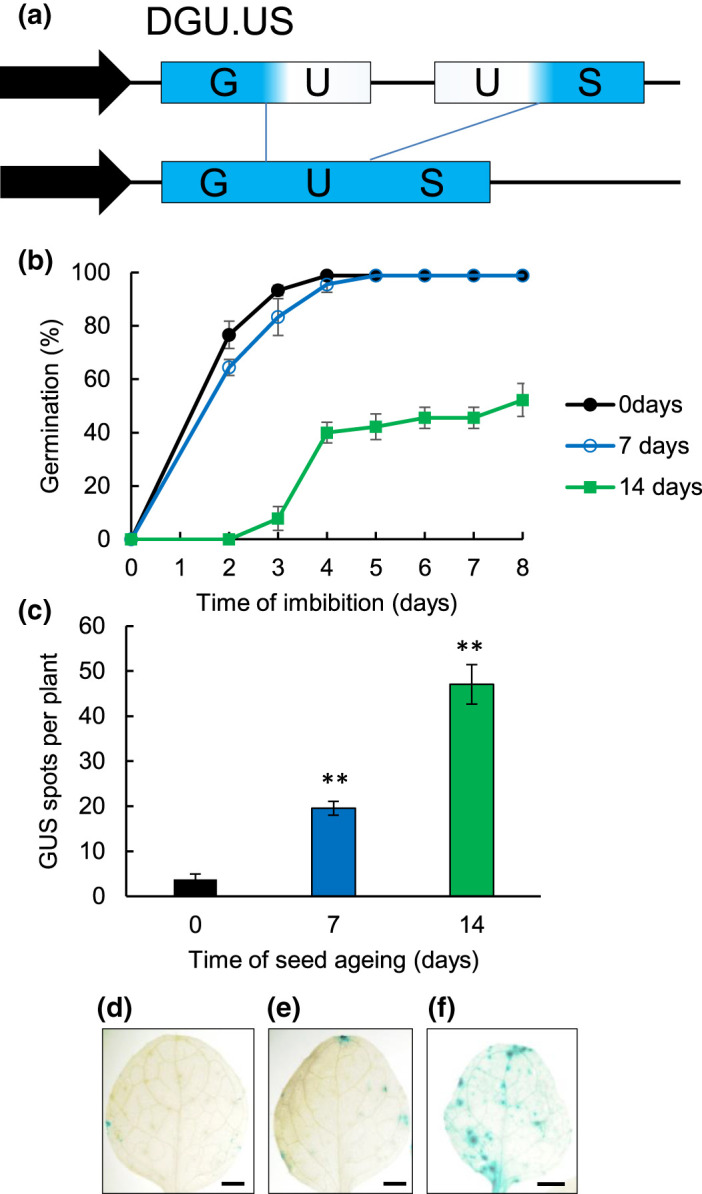
Genome stability in aged seeds. Genome stability was assessed using the intrachromosomal recombination marker line DGU.US which consists of two overlapping fragments of the *GUS* gene with 557 bp of direct repeat (**a**) [16]. Recombination between the GUS fragments results in full length *GUS* and GUS activity. (**b**) Germination performance of DGU.US recombination reporter line after 0 day, 7 days and 14 days accelerated ageing at 35°C and 82%. Seeds were plated and stratified at 4°C for 48 h before transfer to an environmental growth chamber at 22°C and seeds scored for radicle emergence each day post-imbibition. Error bars show the standard error of the mean of three replicates of 50 seeds. (**c**) Frequency of spots and sectors in plants germinated from DGU.US reporter line after 0 day, 7 days and 14 days accelerated ageing. Seeds were stratified at 4°C for 48 h before transfer to soil. (**d**–**f**) Representative images of GUS-stained leaves taken from plants after ageing for (**d**) 0 days, (**e**) 7 days and (**f**) 14 days.

Assessment of genome stability was performed by quantifying intrachromosomal recombination frequencies. The reporter line pDGU.US carries 5′ and 3′ regions of β-glucuronidase (GUS) with an overlapping 557 bp direct repeat which, upon recombination, results in reconstitution of a functional *GUS* gene [18] (Figure 4a). As previously reported, control pDGU.US plants demonstrated low levels of blue sectors (1–5 per seedling) in around half the control plants (Figure 4). Recombination frequencies notably increased upon seed ageing, with a 5-fold increase (*P* < 0.01) as seed lots begin to lose vigour, and increasing to 12-fold increase in spot numbers (*P* < 0.01) as seed viability is compromised (Figure 4b–f). These results are consistent with significant genome instability induced by seed ageing, and support early cytogenetic analysis of the loss of seed longevity.

## Discussion

Germination performance is a major determinant of seedling establishment, with significant impact on crop yield and plant survival in natural ecosystems [7]. Seed quality and seedling establishment are particularly vulnerable to environmental fluctuations arising from changing climates [1]. The seed stage of the plant lifecycle is associated with high levels of genome stress which increases as seeds deteriorate [19]. However, the impact of this damage on subsequent plant growth and genome stability remains little characterised to-date. Here we identify that mutants defective in chromosomal break repair display reduced seedling vigour after natural seed ageing. Moreover we reveal that seedlings germinated from aged seeds additionally show elevated levels of recombination and mutagenesis, likely to impact on plant genome stability. Increased recombination frequencies may be indicative of activation of DNA damage response pathways, or genome instability that results from formation of di-centric chromosomes, a potential product of NHEJ repair. During mitosis, di-centric chromosomes can form anaphase bridges where centromeres are partitioned into a different daughter cells, as observed after seed ageing in multiple studies [12, 20]. Seedlings germinated from aged seeds display elevated levels of PCD in the root meristem that persist through to the early stages of seedling growth. Collectively, this provides evidence of prolonged genome stress post-germination which is a likely contributing factor to the low growth vigour and poor establishment of aged seeds. These results provide new evidence for the importance for DNA repair mechanisms in seeds and seedlings for plant growth and development in the recovery from natural seed ageing.

**Figure 5.**
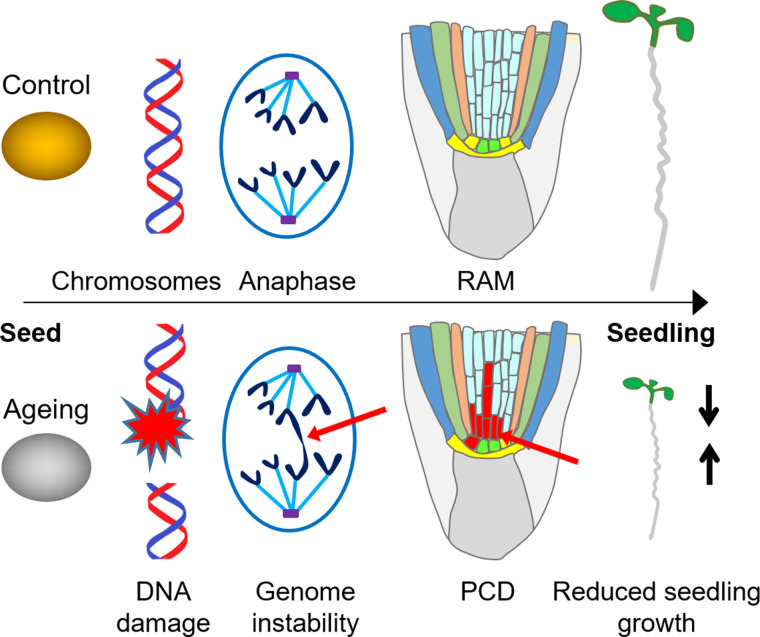
Genome stress and seedling vigour. Summary of seedling responses to genome damage accumulated in seed ageing. Seed ageing is associated with increased levels of DNA damage including chromosomal breaks [3]. Seedlings germinated from aged seeds show decreased genome stability (Figures 3, 4) and increased mitotic defects [12]. PCD in the root apical meristem (RAM) persists throughout seedling establishment in seedlings germinated from aged seeds, in particular from NHEJ mutants, and aged seeds produce low vigour seedlings (Figures 1, 2).

Our previous studies identified established that the seed stage of the plant lifecycle is associated with high levels of genome stress and revealed important roles for chromosomal break repair and response mechanisms in seed longevity [3]. DNA damage in seeds is extensive leading to chromosomal aberrations visible in ∼1% of anaphases at the cytological levels and rising ∼3-fold in low vigour but viable seeds, with similar frequencies reported in a range of crop species [8, 12]. Here, quantitative analyses of genome instability in seedlings germinated from aged seeds was performed using established reporter lines [17, 18]. Quantification of ageing-induced microsatellite instability identified a 2.5–3-fold increase in frameshift mutations in seedlings germinated from low vigour seed lots. The increasing incidence of mutations observed in seedlings germinated from aged seed may reflect both increased levels of DNA damage, decreased repair capacity and impaired DNA damage signalling, arising as a consequence of cellular deterioration (Figure 5) [20, 21]. Similarly, intrachromosomal recombination frequencies increased dramatically as seed germination vigour declined. Stresses result in elevated plant recombination frequencies which may promote adaption and influence plant genome stability at the population level [22].

We previously identified that imbibed Arabidopsis seeds display high resistance to the effects of DNA damage, arising from combination of low cell cycle activity, cell cycle checkpoints and DNA repair that reduces meristem disruption and programmed cell death. However, here we show that the effects of seed ageing on genome stability persist through to seedling establishment. Until the emergent seedling has developed root and shoot systems and become autotrophic, plants in the early stages post-germination are particularly susceptible to multiple abiotic and biotic stresses. Germination vigour determines the success of seedling establishment and lower vigour seed give rise to young plants with increased rates of mortality, negatively affecting crop yields [7]. These early stages of the plant lifecycle are therefore vulnerable to climate change and threaten food security. In addition, natural plant populations need to adapt to rapidly changing environmental conditions including warming and drought in order to maintain ecosystem fitness [23]. Our findings highlight the increased incidence of mutagenesis in aged seeds, including significantly elevated levels of recombination and indels, with important implications for long-term seed storage and genetic stability of plant populations under changing climates [1]. Furthermore, understanding the DNA repair processes important to seed germination performance and viability has potential applications in the development of predictive markers for seed lot quality and genetic improvement of crop resilience to seed stresses. For example, lines with enhanced repair capacity would be predicted to display increased germination vigour and improved, stress-tolerant seedling growth, thereby promoting successful seedling establishment and improved crop yields even in suboptimal climate conditions.

## Materials and methods

### Plant material and growth conditions

Arabidopsis plants were raised in growth chambers under constant humidity (30%), with 16 h light and 8 h dark cycles at 23°C. Arabidopsis Col-0 and mutants were obtained from Nottingham Arabidopsis Stock Centre (NASC). Plants were grown on half strength Murashige and Skoog Basal Medium (MS), 1% sucrose, 0.5 g l^−1^ MES and 0.8% plant agar (Duchefa) pH 5.7 on 16 h : 8 h light dark cycles at 22°C. For each experimental replicate, seeds from all lines were harvested simultaneously and stored at 15°C and 15% humidity for 2 months to allow after-ripening. For natural ageing experiments seed harvests dated from 2012 and 2020 were stored at ambient temperature and humidity. Germination tests and accelerated ageing performed according to published protocols [3, 24, 25] and mean germination time was calculated as described previously [26]. Arabidopsis lines were as previously described *ku80-1* [12]*, lig4-5, lig6-1* and *lig6-1 lig4-5* [3].

### β-Glucuronidase assay

β-Glucuronidase assays were performed on plant tissues fixed for 1 h in ice cold 80% acetone and then incubated for 6 h at 37°C in GUS staining solution (10 mg/ml 5-bromo-4-chloro-3-indolyl β-D-glucuronide cyclohexylammonium salt in dimethlyformamide diluted 1 : 20 in 50 mM sodium phosphate buffer pH 7.0 containing 0.1% triton X-100, 1 mM potassium ferrocyanide and 1 mM potassium ferricyanide). Colour development was stopped by transferring samples to 70% ethanol.

### Statistical analyses

Data were analysed using R. KS tests (*P* > 0.05) were used to test for normality and homogeneous variance was determined using Levene's test (*P* > 0.05). Student's *t*-test were used for pairwise comparisons and analysis of variance (ANOVA) with post hoc Tukey tests was used for multiple comparisons.

### PCD

Programmed cell death was analysed by confocal microscopy using a Zeiss LSM 880 after staining with propidium iodide (10 mg/ml). The *z*-section with maximal cell death was used for quantification of cell death area using the microscope operating software (Zen, Zeiss).

## Data Availability

Data and biological resources presented in this manuscript will be made available upon request.

## Open Access

Open access for this article was enabled by the participation of University of Leeds in an all-inclusive *Read & Publish* agreement with Portland Press and the Biochemical Society under a transformative agreement with JISC.

## Abbreviations

DSBs: DNA double strand breaks
GUS: β-glucuronidase
LIG4: LIGASE 4
LIG6: LIGASE 6
MS: Murashige and Skoog Basal Medium
NHEJ: non-homologous end joining
PCD: programmed cell death
PI: propidium iodide
RH: relative humidity
